# “If I die for touching him, let me die”: a rapid ethnographic assessment of cultural practices and Ebola transmission in high-risk border regions of Tanzania

**DOI:** 10.1186/s12889-024-19316-w

**Published:** 2024-07-09

**Authors:** Priscilla Kusena, Emmy Metta, Hussein Mohamed, Deodatus Kakoko, Tumaini Nyamhanga, Shalini Bahuguna, Nathanael Sirili, Jonas Kinanda, Awet Araya, Alice Mwiru, Stanley Magesa, Lulu Makene, Allan Rwechungura, Fatimata B Kirakoya, Jaliath Rangi, Neema Kileo, Jerry Mlembwa, Method Kazaura, Chipole Mpelembe, Gasto Frumence

**Affiliations:** 1 UNICEF, Plot 133 Karume Road, Oyster Bay, P.O. Box 4076, Dar es Salaam, Tanzania; 2 Department of Behavioral Science, School of Public Health and Social Sciences, Muhimbili University of Health and Allied Sciences, P.O. Box 65015, Dar es Salaam, Tanzania; 3 Department of Environmental and Occupational Health, School of Public Health and Social Sciences, Muhimbili University of Health and Allied Sciences, P.O. Box 65015, Dar es Salaam, Tanzania; 4 Depatment of Development Studies, School of Public Health and Social Sciences, Muhimbili University of Health and Allied Sciences, P.O. Box 65015, Dar es Salaam, Tanzania; 5 Department of Sociology and Anthropology, University of Dar es Salaam, P.O. Box 35043, Dar es Salaam, Tanzania; 6 WHO, Luthuli Street, P.O. Box 9292, Dar es Salaam, Tanzania; 7 Department of Epidemiology and Biostatistics, School of Public Health and Social Sciences, Muhimbili University of Health and Allied Sciences, P.O. Box 65015, Dar es Salaam, Tanzania; 8 President’s Office - Regional Administration and Local Government (PO-RALG), Government City- Mtumba, TAMISEMI Street, P.O. Box 1923, Dodoma, 41185 Tanzania

**Keywords:** Ebola virus disease, Ebola outbreak, Transmission, Cultural practices, Risk regions, Tanzania

## Abstract

**Background:**

Ebola Virus Disease (EVD) is a rare but contagious disease caused by Ebola Virus (EBOV). The first Ebola outbreaks were reported in the Democratic Republic of Congo (DRC) before subsequent reported cases in Western and East African countries, including Uganda, which borders Tanzania. Proximity to EVD-infected countries raises the prospect of cross-border transmission, raising alarm in Tanzania. This study aimed to explore the cultural practices likely to prevent or escalate EVD transmission in the event of its outbreak in the country.

**Methods:**

This rapid ethnographic assessment employed observation, interviews, and focus group discussions to collect data from people with diverse characteristics in five regions of Tanzania Mainland namely, Kagera, Kigoma, Mwanza and Songwe regions and Zanzibar Island. The qualitative data was then subjected to thematic analysis.

**Findings:**

Cultural practices may escalate the transmission of EVD and hinder its prevention and control. These cultural practices include caring sick people at home, confirmation of death, mourning, and body preparation for burial. Communal life, ceremonies, and social gatherings were other aspects observed to have the potential for compounding EVD transmission and hindering its containment in case of an outbreak.

**Conclusion:**

Cultural practices may escalate EVD transmission as identified in the study settings. As such, Risk Communication and Community Engagement (RCCE) activities should be interventionist in transforming cultural practices that may escalate the spread of EVD as part of preparedness, prevention, and control efforts in the event of an outbreak.

**Supplementary Information:**

The online version contains supplementary material available at 10.1186/s12889-024-19316-w.

## Introduction

Ebola virus disease (EVD), formerly known as Ebola haemorrhagic fever, was named after the Ebola River in the Democratic Republic of the Congo (DRC), where the first Ebola case was reported in 1976 [1]. Though rare, the Ebola virus is severe, contagious and highly fatal in humans. Between 1976 and 2018, the reported case-fatality rate was between 40 and 100 per cent [2]. The aetiology of Ebola virus (EBOV) is thought to be linked to fruit bats (family *Pteropodidae*) that serve as a reservoir of the EBOV. The bats spread EBOV to chimpanzees, gorillas, and monkeys, with a subsequent spill over potential to humans. Human-to-human transmission occurs via blood, body fluids, contaminated objects, handling of corpses during funerals, and even by sexual transmission after recovery [3, 4].

Several Ebola outbreaks have been reported in some African countries [4, 5] with the 2014–2016 West Africa epidemic being the most devastating [6]. EVD recurrence in Africa raises concerns that demand answers regarding the potential prevailing cultural contributors to community vulnerability and preparedness for responding to such outbreaks. The most recent EVD outbreak was the 2022 bout of the Sudan Ebola Virus Disease (SUDV) that occurred in Uganda. On 20th September 2022, Uganda’s health authorities declared an outbreak of EVD caused by SUDV identified in 1977. Since then, there have been seven such SUDV infection outbreaks, three in Sudan and four in Uganda. Countries bordering Ebola-struck nations are often at high risk of EVD transmission. As such, East African countries such as Tanzania are at high risk of EVD spill-over via the often-porous border with frequent crossings.

Immediately after Uganda confirmed EVD cases, the Tanzanian government issued an alert, requesting all regions in the country to take precautions to guard against the Ebola outbreak. Specifically, the government identified the border regions of Kagera, Mwanza, Mara, Geita, and Kigoma as the most at risk of EVD spread. The other EVD at-risk regions are those with large airports and bus terminals such as Dar es Salaam, Arusha, Kilimanjaro, Songwe, Mbeya and Dodoma through which passengers from neighbouring countries arrive and transit while mingling with local populations. Such lurking danger necessitates instituting effective EVD outbreak preparedness and countermeasures for timely detection and containment should there be an outbreak.

Like other epidemics, the EVD emergency requires rapidly generated data input to inform the planning and contextualization of interventions to execute effective and appropriate actions. Evidence suggests that enhancing public health intervention efficiency requires a thorough confirmation of the effectiveness of interventions aimed at changing behaviors and their determinants as well as how they impact health outcomes [7, 8]. In this regard, knowledge, attitude, and practice (KAP) studies assess the awareness and knowledge about a particular disease. Several KAP studies on EVD have been conducted in high-risk countries [4, 9, 10], with little attention paid to non-high-risk or low-risk countries. Consequently, anthropological data on EVD vulnerability and response preparedness in countries like Tanzania have primarily been lacking. Hence, social science data must be gathered and translated in real-time to support planning and preparedness interventions for the prevention and control of the EVD transmission should the need arise.

After all, multiple interventions and cooperation from various stakeholders are required to treat and prevent EVD and halt its further transmission. These measures include surveillance, infection prevention and control practices, case management, contact tracing, community engagement, social mobilisation, safe burials, and good laboratory services [2, 11]. Besides strengthening the health systems, cultural factors at the community level that characterise different exposures to diseases need consideration when designing any preventive interventions against disease transmission and outbreaks. Empirical literature attest to how cultural beliefs and practices are pivotal in spreading diseases including the dreaded EVD [12, 13]. As such, a social anthropological study can generate in-depth data essential in understanding the cultural risk factors and mechanisms for disease transmission to inform interventions, management, and control. Therefore, this study sought to explore the cultural practices likely to prevent or escalate EVD transmission in the event of its outbreak in the country. The findings of this study provide important insights for informing culturally sensitive preparedness efforts to prevent further EVD transmission and its escalation should an outbreak be imminent.

## Methods

### Study design

We adopted a rapid ethnographic assessment to explore the cultural practices likely to prevent or escalate EVD transmission in the event of its outbreak in the country. Ethnographic design was deemed appropriate as EVD transmission and prevention strategies are affected by cultural practices. We employed observations, in-depth interviews, key informant interviews, and focus group discussions to collect data. We adopted the grounded theory principles of constant comparison [14, 15] to investigate social processes influencing EVD vulnerability in the cultural context of the research sites and facilitate documentation of the common patterns across participants and study settings. We started with an observation, analyzed information, and made sense of what we saw. Then the emergent learning decisions informed the where, how, and from whom to collect data for the study. In essence, the application of grounded theory principles systematically guided the research teams’ probe focus, structure, and organization of the research process. Such an approach also produced analytically prioritized cultural issues while addressing other secondary dimensions.

### Study context

The study was conducted in purposefully selected EVD high-risk regions of Tanzania’s Mainland comprising Kagera, Mwanza, Kigoma, and Songwe [16]. Both the Kagera and Mwanza regions border Uganda, which has experienced several recurrent EVD outbreaks. Similarly, Kigoma and Songwe regions share borders with the Demographic Republic of the Congo (DRC), which has also experienced frequent EVD outbreaks. The study also covered the Zanzibar archipelago consisting of the Isles of Unguja and Pemba. Local and international travellers, including those from EVD-affected countries, could travel to these islands as part of the United Republic of Tanzania. For a detailed description of the study context see Annex 1.

The selection of the specific data collection sites from the study regions was participatory since the process involved regional and district health authorities. The criteria for selecting the sites entailed identifying areas with indigenous people capable of describing critical cultural issues that compound the EVD transmission risk in case of an outbreak.

### Data collection team

The data collection team comprised of research assistants with a background in social sciences and a major in anthropology; they also had experience in health-related ethnographic fieldwork activities in rural and urban Tanzania. This team worked collaboratively with six researchers in data collection. In addition, the data collection team benefited from data collection training covering the study background and its objectives, Ebola transmission and prevention, cultural practices related to Ebola transmission, study methodology, and data collection guides. The training was followed by pretesting of the data collection guides and their refinement before actual use in the study.

### Data collection methods

We employed a series of data collection approaches using data collection tools developed [16]. Initially, non-participant observation allowed us to identify EVD risk of transmission areas, specifically those with high human interactions due to economic as well as sociocultural activities. The observation findings informed the purposive recruitment of participants for in-Depth Interviews (IDIs), Key Informant Interviews (KIIs) and Focus Group Discussions (FGDs), see additional file 1 on the participants recruited per the respective data collection method.

### Observation

Observations were the main data collection methods, and they were conducted to understand the real-life behaviors and risk contexts of EVD transmission in communities. The study settings included hospitals, markets, funeral yards, ports, bus terminals, borders, airports, churches, mosques, and schools. These observations provided first-hand information on the actual behaviors in natural settings and helped determine preparedness for preventing and combating EVD. The observation process was iterative, with repeated observations at some sites. The observations were complemented by informal conversations with community members to identify issues requiring further insights. The observation method was complemented by other data collection techniques, such as IDIs, KIIs, and FGDs, to gain comprehensive insights into EVD transmission.

### In-depth interviews

In-depth interviews (IDIs) were conducted with prominent community members, including religious leaders, retired civil servants, and journalists. The purposive interviews allowed exploring multiple angles of the issue under consideration. We adopted the maximum variation sampling to facilitate documentation of unique or diverse variations and identification of the common patterns across participants and study settings [17]. We attained information saturation at the 53rd IDI where no new information was coming out with more IDIs. We analysed the data concurrently with those from KIIs, Observation, and FGDs to establish the tentative categories, which prompted us to conduct theoretical sampling to understand and enrich further the tentative categories. With theoretical sampling, we stopped data collection at the 66th IDI where no further information was coming out regarding the tentative categories. Both the initial and theoretical sampling IDIs were conducted in areas convenient for the participants and which ensured privacy. All the interviews lasted between 45 and 60 min and were digitally audio-recorded.

### Key informant interviews

We purposefully selected key informants based on their positions and roles in the communities involved in this study. These included village executive officers, regional medical officers, bus-stand managers, port health in-charges, and health facilities. The interviews aimed to grasp cultural practices and their impact on EVD transmission and prevention. The interviews were conducted in a quiet, comfortable place and were audio-recorded with prior consent. The interviews helped to enrich and understand the findings by complementing data from observations and IDIs while focusing on how cultural practices could shape EVD transmission and inform prevention. The first round of KIIs ended after the 44th informant after attaining information saturation where no new information was coming. After analysing them together with observations, IDIs and FGDs, we developed tentative categories. To come up with the meaning grounded in the data, we continued theoretical sampling and interviewed further 13 key informants and we stopped further interviews at the 57th participant as there was no further explanation to the tentative categories that was coming out with continued interviews. The KIIs lasted between 30 and 60 min and were audio-recorded with prior permission.

### Focus group discussions

Focus Group Discussions (FGDs) were conducted with adult community members, including fishermen, traditional healers, youths, pastoralists, commercial sex workers, and *bodaboda* (motorcycle-for-hire) drivers. The discussions focused on community values, myths, beliefs, cultural norms, and practices related to taking care of patients, managing corpses and burial. The FGDs were conducted in a quiet and convenient locations and lasted between 55 and 90 min. The homogeneity of each group was maintained to enhance the discussions.

### Data analysis

The study used grounded theory principles for data collection and analysis. Additionally, we held daily debriefings and summaries to identify key themes for further follow-up in subsequent data collection activities. Observational findings guided sampling for IDIs, KIIs, and FGDs. Data analyses were stepwise, with a codebook developed based on the study objectives. The analytical approach used both deductive and inductive strategies.

The audio-recorded files were transcribed verbatim after data collection, and analysed using open, axial, and selective coding methods [17]. The code book used in the study was based on the five transcripts picked from each study region and was finalised after consensus among the researchers. The emerging new codes were then listed, discussed, and agreed upon as new or as part of the existing codebook. The process began with open codes, followed by sorting and grouping of similar codes in team meetings. Axial coding began after grouping similar codes and renaming some. After axial coding, we developed several sub-categories and with selective coding we grouped them into four categories or themes. The process entailed identifying and describing the central phenomenon or generic categories within the data that best captured the perspectives of the study participants [17, 18]. The main emerging categories or themes were care of the patients and EVD transmission risks; death, mourning and preparation of the body and EVD transmission risk; communal way of life and EVD transmission risks; ceremonies and social gatherings and EVD transmission risks. Even though the cultural practices reported in the study were reasonably consistent, the findings are not presented separately by participant type; instead, the descriptions are under different themes pertaining to EVD transmission risk as they emerged from the data.

### Findings

#### Care of the patients and EVD transmission risks

The study findings indicate various risky practices related to the care of patients, which could transmit Ebola. During interviews, participants reported practices such as unprotected physical contact when caring for sick persons even when the caring took place in a health care facility where protective equipment would be expected. Indeed, taking care of or supporting the sick while wearing protection such as gloves was reported as an uncommon practice across study sites. The participants reported that even when one had the protective gear, they would still visit and attend to the patient without using them as this participant explains:

*… when there is a sick person or a tragedy, it is not until you are invited … when you hear it, you go to comfort your fellow… sometimes, you have the gloves with you, but you visit a sick person at home, you attend to him/her without using them, you remember that you did not use them on your way back home…* (Zanzibar, KII0 6)

The participants further reported non-use of protection equipment when caring for a sick person at home fearing it would tell the patient that they are very sick or instigate fear among family members. The study informants indicated that people in the community believed that everyone would die at some point, whether they used protection or not; this belief eroded the prospect of using protective measures in situations of disease outbreaks. They also reported that the idea of wearing gloves, for example, when carrying and supporting a sick person did not easily enter the caregiver’s mind. When the caregivers must do so, they tend to refuse, with some saying, “If I die for touching him, let me die.” When explaining this one of the KIIs said:

*A caretaker may say, “My child is suffering, and they say I should not touch him! So, if I die for touching him, let me die… You see… she will continue to take care of him without taking any precaution due to the belief that everyone will die. That thing [use of protective gloves] does not exist because people have a belief that everyone will die even if you do not touch a sick person* (Bukoba, KII 010)

Participants reported that the non-use of protective gear when caring for a patient also resulted from a fatalistic attitude, believing that everything is pre-determined and that they have no control over their destiny. Implicitly, to them one’s fate about the risk of being infected and dying was God-given, hence rendering what one does to protect himself or herself immaterial. In this regard, one of the participants in Kigoma said: *“If it is not your time to die nothing will happen to you, even if you do not use the gloves”* (Kigoma KII03).

Furthermore, the study also observed gender dimensions of vulnerability. During observations at the healthcare facilities, caregivers were largely women. Across the study sites, it was observed that women mostly cared for their families, including during illness. Women reportedly cared for the sick family members at home and those hospitalized. Such care practices could entail feeding the sick person, bathing him or her, and washing clothes. Participants reported that these care activities aggravated women’s risks of contracting EVD in the event of an outbreak. During interviews, one of the participants explained that these gender roles and practices are grounded in the community’s cultural norms:

*Traditionally, in our society, there is a father, mother, children and relatives. Most often, when there is a sick person, the one who takes care of the patient when at home or admitted at hospital is the mother because she is a housekeeper, she takes care of the family, and the father must go out to work and earn money to support the family (*Bukoba, IDI07)

#### Death, mourning, and body preparation for burial and EVD transmission risks

Participants further reported that during the dying moment, the process may pose risks to Ebola virus transmission if the victim dies of the disease. They specified that people who witness the dying moment of a person commonly struggle to save the person’s life. In many cases, they tended to touch the chest of the dying person to determine whether the person was breathing, or the heart was still beating. This process involved pressing the dying person’s hands to test whether the blood was flowing through the veins or touching different body parts to feel the body’s temperature due to the common belief that a dead body was unusually cold. Furthermore, after a person dies, people close the deceased’s eyes and mouth and straighten the body posture. The respondents reported these acts as an essential cultural practice executed to confirm the person’s demise and ensure the body was well-postured in readiness for burial:

*When a person is dying, you find the people around trying to save the dying person’s life…you see them touching the chest, checking for temperature, and touching legs to see if the person is cold because of the belief that a dead person’s body is cold. After dying, they close the dead person’s mouth, straighten his/her hands and legs, and ensure the body is well-postured, this touching of the body can present high risks for infection in the events of EVD. (*Mwanza, KII09*)*

Participants in the interviews and focus group discussions also explained that these processes ensure the person dies in a dignified manner, “humanly.” Moreover, culturally the widespread belief was that ignoring caring for the dying person might subject one to similar neglect or the ghost of the deceased could return to haunt them. The notable heightened physical contact between people milling around the dying person also exposed them to the transmission of the disease that had occasioned the death in the first place. During the discussions, participants commented that physical contact that happens at the dying moment could transmit the Ebola virus once the patient dies of EVD.

Participants further reported that mourning starts soon after the announcement of the death. The mourning practices reported in this study entailed sitting near each other with inevitable physical contact including hugging and shaking hands among mourners. Communal eating and sleeping over at funerals were also reported as standard mourning practices and are perceived to compound the risk of EVD transmission:

*…. relatives of the deceased converge and sleep in the house of the deceased; they typically crowd in the house of the deceased or the deceased’s relatives where the mourning is happening, stay and sleep there for several days as a way of expressing concerns for the departed person and showing love and togetherness to mourners. So, you see for yourself, in the event of EVD it will be dangerous in that way …* (Mwanza, KII 10)

Observation affirmed that during mourning it is customary to hug and shake hands with bereaved family members. To the participant, this was a culturally significant practice as it signified closeness between people and was a sign of expressing regret for the loss:

*At funerals, based on our culture, you have to hold his hand, or he holds you like this [gesture] sorry, sorry for the loss, if someone has the disease, you may contact it easily even through tears left on the hands (Bukoba*, FGD010 Participant 3)

Not doing so in the areas under review might result in other mourners suspecting the person of being an evildoer or someone insensitive to social etiquette. In fact, during observations in different settings, handshaking and hugging emerged as standard practices. This practice appears to be embedded in religious and traditional norms and values. It was also evident that many people shake hands and hug when greeting each other, during prayers and social gatherings such as meetings and weddings, and when comforting each other during difficult times.

Regarding preparing the bodies for burial, when a family cannot pay for mortuary services offered at the health facilities, they preserve the body traditionally at the deceased’s home by applying local/cultural means, such as the application of salt, honey, and burning of herbs near the body. Sometimes, people place banana leaves on the ground, lay the body over, and cover it with other banana leaves. The executors of these deeds lack protection and safety gear. Such practices devoid of safety precautions emerged to have the potential of escalating EVD transmission because people engage directly in preserving the body of the dead:

…*if he is dead…. The mattress will be removed…. They will put him on the bed without a mattress. I often see that they apply salt on the dead body so that he does not smell.* (Mwanza, KII02)

#### Communal way of life and EVD transmission risks

The study also observed that across sites community members lived communally. The obvious sign noted was sharing that appeared engrained and synchronized in the norms and customs of the communities as practiced in many facets of their daily life. For example, during observations, it was common to see many people washing their hands using the same bowl of water before they ate. People were soaking their hands in a single bowl, sometimes with or without soap, and there was no running water. When this was pursued during the interviews, one of the participants reported that is a way of life in rural settings and that the practice is okay, it doesn’t have any problems:

*We do that always, there is no problem. That is the life in the village*” (Mwanza. IDI011)

During observation, it also emerged that sharing was a common feature during eating and drinking practices. Groups of people ate and drank together on the same plate or bowls to show love and care for each other. In the Kagera region, cultural practices that favor many people eating together from a single plate or on banana leaves, “embabi”, was reported to be a common feature in many households to symbolize peace and love among the people as part of their collective well-being. Across the study sites, participants reported that frequently, people placed a bucket full of water for drinking and a cup nearby that they heartily shared as they ate. Sometimes, family members imbibed the water from the cup and returned the remaining water and the cup to the bucket from which another person drank using the same cup. Although such practices could escalate EVD transmission, they also serve important roles in the community. Participants indicated that the shared eating and drinking habits fostered unity and dispelled distress and selfishness in communities. Moreover, parents had an opportunity to teach their children good manners and culturally acceptable conduct within these households and the community in general. On the other hand, eating and drinking from the same plate and cup, respectively, was reported to be common not only at the household level but also during ceremonies and big gatherings:

…*ceremonies that involve gatherings of many people, they normally eat together using a big plate, known as “sinia” in Kiswahili or banana leaves (embabi) as people sit around it and eat together* (Missenyi, IDI0112)

Participants indicated that, culturally, eating together signalled unity, love, and solidarity. As this participant from Zanzibar explained:

…*Brother we are hustlers, we go out daily to earn our daily bread…eating together is a blessing and it brings love, that is Islam…But if it comes as a government order that do not share bed, do not eat together…. as there is an outbreak, we will obey…* (Zanzibar, IDI04)

Furthermore, the participants reported that people eat together from the same plate to ensure everyone had a share of the food. In some discussions, the practice of sharing plates during meals was mentioned as a risk that could expose the many people sharing it to infections in the face of an epidemic because when eating some people lick their fingers and touch the food, hence facilitating transmission of disease-causing viruses, especially in the context of viral epidemics such as EVD.

Sharing was also reported to be a characteristic in sleeping patterns and use of beddings. Across the sites, sharing of beds and bedding with either family members or friends was reported as a common phenomenon. The practice was mentioned as not only engrained in the cultural milieu of the communities but also as shaped by the real-life situation whereby many community members could not afford beds and bedding for everyone. Sharing of beds and bedding was reported as inevitable due to large families:

*…just imagine in a family of 20 people, and you have only a three-bedroom house, what you do? You have no option but to share the rooms*… (Songwe, IDI05)

It was also reported that this kind of sharing became aggravated when families received visiting relatives during holidays, weddings, and funerals. Most of the households reportedly had three bedrooms, one for parents, one for girls, and the other one for boys. The girls’ and boys’ rooms accommodated all the female and male visitors, respectively:

*You find that a family had visitors and all sleep [crammed] in the boys for men or girls for women rooms. It is common to see several of them sharing the room…. several sharing the mattress and others sleeping on mats…. this is dangerous for the transmission of diseases (Mwanza, KII016)*

This sharing of the bed and bedding occurred both within families and when people were in temporary settlements such as in fishing sites. Fishing sites attract people from different areas for fishing and small business activities, who normally form groups and work as friends. These groups build small huts where they sleep after their fishing activities. They stayed in these huts during working days and vacated to link up with their families during weekends:

*…for instance, look at this hut. You will see a fishing boat with around 20 people who have gone out for fishing and when they come back they will enter and sleep in there…during the weekend they go to their families and return here after the weekend…”* (Zanzibar, IDI07)

During observations, fishing communities lived in small huts. The overcrowding covered people of different ages and genders and socioeconomic characteristics. People sat in groups and their business operated in crowded areas. The participants insisted that such overcrowding was a recipe for disaster since it heightened the risk of the populace contracting infections.

#### Ceremonies and social gatherings and EVD transmission risks

Participants reported that some social meetings were invariably attended by many individuals. Social events such as weddings, harvest festivals, kitchen parties, sports and games, public events (public meetings, memorials days) and cultural ceremonies such as female genital mutilation and male circumcision ceremonies usually attracted large crowds. These events reportedly attracted people from within and outside the study communities including from the neighbouring countries with a history of EVD attacks. Such a context reportedly exacerbated the risks of EVD transmission:

*“During send-off and wedding ceremonies, many relatives and other invitees including friends and relatives from neighbouring countries like DRC, gather in a hall and this is a risk for Ebola transmission”* (Songwe, FGD01 Participant 3). Wedding celebrations have been linked to an increased risk of Ebola transmission because many individuals, particularly relatives and friends, congregated in one space. Numerous activities during the celebration such as embracing one another, shaking hands during greetings, and dancing together, carrying a danger of spreading the Ebola virus. Several wedding ceremonies were observable during fieldwork; indeed, crowds of people were evident in all research regions. Such a background was reported to raise the possibility of spreading diseases such as Ebola in events of outbreak:

*When we organise the wedding, family members build tents or hire a venue and invite many family members and friends. We all converge in a congested hall. Sometimes, we may be four up to six hundred in one hall, we drink and eat together. Such gathering is huge and a risk for Ebola transmission* (Missenyi, IDI 8,)

Generally, the study findings indicate that shaking hands and hugging were normal and socially valued practices during ceremonies and social gatherings. Such practices are deep-rooted in the socialization process and culturally sanctioned as means for expressing care, love, and togetherness in gatherings such as funerals, weddings, religious masses, and festivals.

## Discussion

This rapid ethnographic assessment aimed to explore the cultural practices likely to prevent or escalate EVD transmission in the event of its outbreak in the country. The analysis revealed that communal life, ceremonies, and social gatherings have the potential to escalate EVD transmission and hamper EVD control in case of an outbreak. The communal life is grounded in common sociocultural practices such as caring for sick people at home, confirmation of death, mourning, and body preparation for burial.

Practices of caring for sick people at home as revealed by our study findings, emphasize the value of community care and do not promote stigma as they are based on local customs and reinforced in the communities’ daily life. However, such practices fuel the transmission of infections, including EVD [19, 20] and other epidemics [21]. Given the EVD transmission nature, concerted efforts aimed at guiding family caregivers on safer practices when caring for their sick relatives at home as part of embracing their cultural norms and values are inevitably needed.

Furthermore, from our study findings, the practices of caring for a sick person at home was engendered. In these communities, it was a cultural standard that men are expected to work outside the house and women to work in the households. Among the activities that women are tasked with include providing care to family members, including care of the sick, activities such as feeding and bathing the sick, washing, and general cleanliness. Such practices exposes women to more infectious diseases, including EVD, than their male counterparts [3]. The gendered infection risks are inherent in the power structures of many of African countries and, ultimately, perpetuate woman’s vulnerability to disease while fulfilling their culturally enshrined roles [22]. Incidentally, social norms and values require women to shoulder care provision responsibilities with less attention paid to capacitating them to perform their roles safely [23]. The situation further met challenges stemming from the prevailing economic inequalities and decision-making powers between men and women cemented in the cultural milieu [24]. Thus, RCCE interventions aimed to address the disparities between men and women in disease vulnerabilities are needed. Moreover, women may be empowered socioeconomically by the Government by making personal protective materials freely available during the disease outbreak or are accessed at subsidized cost.

The study findings further indicate cultural norms and values that characterize death confirmation, mourning, and preparation of the body for burial activities are potential escalators of EVD transmission in the event of an outbreak. The process of touching the dying person and ensuring the death occurs in a dignifying manner are risky practices in the context of EVD. Particularly, EVD virus is mainly transmitted via human-to-human through direct or indirect contact with infected body fluids [1]. Burial-related activities are widely reported as a source of infection transmission in settings with EVD [5, 19, 25, 26]. The danger of infection transmission can be heightened by huge crowds, personal contact, and touching the corpse of the departed at funerals during mourning. People also frequently manage the casket with unprotected or exposed hands. These behaviours raise the possibility of widespread infections in the event of an EVD outbreak in Tanzania because of their strength in favouring disease transmission, including epidemics [27] and complications in disease prevention and control. Previous research [3, 5] demonstrated the importance of funeral and burial customs, including embracing, shaking hands, and crying and mourning in disease transmissions [28]. Efforts to prevent transmission and prepare for EVD responses make RCCE interventions aggressive in raising community awareness on the disease transmission risk embraced in the cultural practices that are cherished in the communities and inculcate the culture of preventing diseases from emerging and spreading [29]. Thus, efforts should also engage communities in identifying better ways to modify their cultural norms and values regarding death confirmation, mourning and preparation of the body for burial to an effective level in preventing diseases yet culturally acceptable.

This study also found that the communal way of life was characterized by the sharing norms and values carry the possibility of escalating EVD transmission during an epidemic. The commonly reported sharing of beds and beddings, clothing, bed linens or utensils was critical in fostering household EVD transmission in Sierra Leone [30]. Similar observations were also reported in Guinea where sharing beds and sleeping mats fuelled EVD transmission [25] and reported from other settings [31]. As EVD is a contagious disease, its risks for further transmission rises with direct human-to-human contact via bodily fluids or indirect contact with a contaminated surface [32]. This is particularly tenable when sharing beds and bedding. As such, the sharing of beds and bedding reported in the study raises major concerns in the context of EVD outbreak transmission and the transmission of other epidemics. This practice suggests the need for continued awareness creation on the risks for household epidemic transamination and adaptation of better sleeping patterns to limit escalation of epidemic transmissions in case EVD, among other epidemics, erupts.

Large crowds and groups were observed with people sharing meals on single plates in line with social etiquette across the study settings. Sharing meals was reportedly standard practices and sign of peace and love in the study. Although literature documents less the role of food handling and consumption on EVD transmission risks [33], food is a reported route for transmission of other viral diseases. Contaminated food can negatively affect individuals after consumption or during food handling, as reported for Hepatitis A [34] and norovirus [35]. Similarly, Lassa fever, the virulent acute hemorrhagic fever, closely resembling EVD, is transmitted to humans via contaminated food or objects [36]. Logically, sharing meals and contaminated food could ease the transmission of EVD in the communities; however, such allegations further need research and analysis. Gaining such understanding could be critical for informing RCCE activities in emphasizing the role of communal life, particularly meal sharing that abets infection transmissions not only in EVD situations but also in epidemics of other infectious ailments.

In the study settings, ceremonies and social gatherings were prevalent. Reportedly, social gatherings are inevitable in the communities, and some of them, such as weddings and harvest festivals, attract people from within and outside the communities’ boundaries, including people from neighbouring countries hard-hit by EVD. Such a background exposes the community members to EVD transmission in the face of an outbreak. Evidence from countries that had EVD outbreaks shows that ceremonies and social gatherings accounted for the rise of incidences of infections [3, 37, 38]. Social gatherings and ceremonies were also implicated in the rising risk of COVID-19 infections across different settings [39–41]. In the study, it emerged that ceremonies and social gatherings characterized by embracing and caressing one another shaking hands and dancing together are high-risk factors for infection transmission. In Ghana, handshaking was commonly practiced as a typical gesture of love and affection at social gatherings [3]. There is also a need to regulate such practices in the context of infectious diseases to prevent transmission and their control, especially should there be events of re-emerging and emerging epidemics.

### Strength and limitations

This rapid ethnographic assessment was conducted in selected high-risk regions in Tanzania. The study design permitted the application of multiple data collection methods and rapid data collection across different EVD high-risk border regions that was much-needed to inform RCCE activities as preparedness for EVD prevention and response should there be a cross-border spill-over of Ebola transmission. The application of different data collection methods enabled data triangulation and strengthened the quality and reliability of the study findings. Although the study was conducted in border regions and other areas identified as “EVD high-risk regions”, the cultural practices reported in this study can also be applicable in EVD low-risk regions because of shared cultural norms and values in Tanzania and other African regions generally. However, some nuanced cultural practices that may escalate EVD transmission may not be generalized in other regions.

## Conclusion and recommendations

The findings underscore that communal life embedded in otherwise acceptable cultural norms has the potential to escalate EVD transmission and hamper its control during the outbreak. RCCE should, therefore, focus on transforming such practices as part of preparedness efforts for EVD prevention and control in the event of an outbreak to ensure they do not become an impediment to intervention measures. Implicitly, the urgent need for culturally adaptive RCCE approaches. As behaviour change strategies take time, efforts to empower the community to adopt safe measures while embracing their cultural norms are inevitably needed as immediate measures during EVD and other outbreaks. One approach could be to inculcate a culture of general infection prevention and control within families and communities by conducting dialogue sessions through Radio and Television programs. The necessity of prompt preparedness can never be overstated, given the recurrence of EVD infections and the risk of cross-border transmissions in the border regions.

### Electronic supplementary material

Below is the link to the electronic supplementary material.

Supplementary Material 1

## Data Availability

Data is provided within the manuscript and supplementary information files.

## Abbreviations

DC: District council
DRC: Democratic Republic of the Congo
EBOV: Ebola virus
EVD: Ebola virus disease
FGD: Focus group discussion
IDI: In-depth interview
KAP: Knowledge, attitude, and practices
KII: Key informant interview
MUHAS: Muhimbili University of Health and Allied Sciences
PO-RALG: President’s Office - Regional Administration and Local Government
UNICEF: United Nations Children’s Fund
RCCE: Risk communication and community engagement
