# Supplementary material for: “If I die for touching him, let me die”: a rapid ethnographic assessment of cultural practices and Ebola transmission in high-risk border regions of Tanzania
Source: BMC Public Health. 2024 Jul 9;24:1838. doi: 10.1186/s12889-024-19316-w (PMC11234704; doi:10.1186/s12889-024-19316-w)
Supplement: Supplementary file 1 — Supplementary Material 1 [file 12889_2024_19316_MOESM1_ESM.pdf]

Rapid Ethnographic Assessment Data collection tools.

1. **<u>Observation (*and informal discussion*) guide in the community/ business places</u>**

    1) **Caring of sick persons at home**
        a) Who is responsible at family level?
        b) Caring by relatives
        c) Description of household management of infectious diseases including both risk and positive practices
    2) **Healthy seeking behaviour**
        a) Traditional practices on health seeking behavior (e.g., first contact when a family member is sick, observe kinds of drugs used traditional vs modern)
        b) Visit traditional healers and observe
            - Kinds of people vising the traditional healers (by sex, estimated age)
            - Physical appearance (e.g., observed clinical signs such as coughing etc)
            - Accompanying personnel (or alone)?
            - Practices such as bathing, sleeping
            - How are drugs given? Drinking, apply on the skin etc
            - Isolation room (practices) if any?
            - Hygiene facilities (hand washing)?
    3) **Burial practices**:
        a) Local beliefs and practices around illnesses and death and the nature of burials
        b) Traditional and religious practices and handling related to pre burial caring of the deceased person
        c) Gathering among mourners
        d) Handling of family members
        e) Handshaking practices
        f) Food/drinking serving?
    4) **Community centers (markets, churches, mosques, informal gatherings)**
        a) Hand wash facilities
        b) Education materials such as posters (on EVD)
        c) Health committees (social welfare)
        d) Religious beliefs and practices on Ebola
        e) People's beliefs about infectious diseases
        f) Handshaking practices
        g) Sharing of glasses in bars and local brew places
    5) **Community information flow patterns**
        - e.g., influential persons trusted to communicate serious and controversial matters in the community
    6) **Ports of entry**:
        a) Characteristics of cross border people
            - Estimated number of people (male vs female)
            - Interaction patterns
            - Drivers (positive and risk behaviours and practices)
            - Their knowledge on EVD preventive measures

b) Screening practices,
c) Use of PPEs,
d) Availability of WASH facilities
e) Availability of information centers
f) Availability of IEC materials
g) Availability of emergency/isolation facilities

7) Present hypothetical cases "scenarios' about EVD
   a) People's beliefs about EVD including their causes, identification of signs & symptoms
   b) Possible vocabulary already developed in the area to talk about dangerous infectious disease
8) Identification of other cultural characteristics and conditions that are likely to influence responses to EVDs or any other infectious diseases including support and care system

2. Focus Group discussion (FGD) /In-depth Interview (IDI) guides

1) What do you understand about disease outbreaks? Probe for:
   a) Examples of disease outbreaks
   b) What outbreaks have occurred in this area?
   c) What do you think was the origin of these outbreaks?
2) What can you say about Ebola disease? Probe for:
   a) What are the main signs and symptoms?
   b) What are the causes of Ebola?
   c) How can it be transmitted?
   d) How can you prevent Ebola?
   e) Who do you think can get Ebola? Why
   f) Who do you think cannot get Ebola? Why?
   g) How do you see yourself in terms of risks to contact Ebola? Why?
3) Normally where do people in this community seek health services when are sick? Probe for all health service sources and who are most likely to consult each?
   a) How common do people in this community consult drug shops for health services?
   b) What are the reasons for consulting drug shops?
   c) How common do people in this community visit traditional healer?
   d) What are the reasons for consulting traditional healers?
   e) How common do people consult spiritual or religious healers when have health problems?
   f) What are the reasons for consulting spiritual/religious healers?
   g) How is the decision made on where seek health services? Who makes that decision?
4) To the best of your knowledge, just in case you encounter a person who is suspected of having Ebola like symptoms:
   a) What would you do?
   b) What do you think your family would do?
   c) How about other people in the community, what will they do?

5) To the best of your knowledge, how best can people suspected of having EBOLA be cared for in their families or communities? (Probe for specific actions at family level and community level)
6) What beliefs/practices do you think can put someone at risk of getting Ebola? Probe for :
    a) Which among the beliefs/practices you mentioned above are present in your community? How can we mitigate them?
    b) What do you think can be done to avoid the risky beliefs/practices?

    (If that person/group cannot identify them, mention them, e.g. Sleeping near a highly infectious corpse for several nights, believing that doing so allows the transfer of power, believing that traditional healers can cure diseases etc.)
7) What norms, traditions and customs you know that can put someone at risk of getting Ebola? Probe for:
    a) Which among the norms, customs and traditions you mention above are present in your community?
    b) How can we mitigate them? **(If that person/group cannot identify them, mention them, e.g. Burial traditions such as some mourners bathe in or anoint others with rinse water from the washing of corpses, sleeping with a corpse inside the house before burial)**.
8) What can be done to prevent Ebola from entering in your community and our country?

    Probe:
    a) At family level, community level and national level)
    b) What can you do?
    c) What support will you require?

9) What roles can other community members do to prevent Ebola?
    a) What can women do?
    b) What can men do?
    c) What can the youth do?
    d) What can religious leaders do?
    e) What can community leaders do?

10) What are the communication channels used to communicate information on disease outbreaks and prevention in the community? Probe:
    a) All (list) existing communication channels (e.g electronic – radio, TV, mobile phones; print – magazines, newspapers, posters/banners, fliers etc.; outdoor – billboards etc.; interactive – roadshows; interpersonal (door to door visits, community meetings, focus groups etc.), social media, internet-email, websites)
    b) Which ones do people trust most? Why? (Consider the different population groups e.g., local leaders, community members, elderly, men, women, youth etc)
    c) Which ones are likely to reach the majority in the community?
11) What are the key messages provided when there is or potential of disease outbreak in the community? Probe:
    a) Disease signs and symptoms, risk sources, how to prevent etc
    b) Who provides that information? How?

c) What do you think misses when such information is provided?
d) What should be done for such information to be useful and beneficial to the community members? Consider the different population groups

12) What are the preferred communication channels and messages to inform communities on Ebola? Probe for:
   a) Messages that work well in the community, that are also cultural and language sensitive and acceptable
   b) What is the best way to deliver those messages to reach the majority especially women?
   c) What is the best language that can be used to reach the majority in your community? (Probe for Kiswahili or local language).
   d) What communication channels are likely to reach the majority in the community? Why?

13) Who would be the best person to disseminate information about Ebola in the communities? Why? Probe:
   a) Teachers
   b) Health care workers
   c) Community leaders,
   d) Youth,
   e) Women etc.)
14) What can stop people from getting clear and correct information about Ebola in your community?
   a) How does that affect communication?
   b) What can be done to address that?
15) Ebola virus disease can be transmitted through eating infected bush meat; contact with saliva, nasal secretions, excreta, faeces, and fomites of infected person; contact with Bats; contact with infected bush meat, mourners who have direct contact with bodies of a deceased Ebola patient, during burial rituals, attending/participating in funeral of a deceased Ebola patient and people who travel to Epidemic Areas. Now think of those means of transmission,
Probe:
   a) Which among those are present in your community?
   b) How best can we educate the communities from avoiding/mitigating those cultural/practices issues?
   c) How can that communication to reach the majority in the communities?
16) What are the main economic activities in this community? Probe:
   a) How are these activities likely to promote transmission of EVD in the community
   b) How are these activities likely to challenge EVD prevention efforts? And what needs to be done to avoid that?
17) How do people in this community cope in case of disease outbreak? Probe:
   a) Existing community structures on prevention of infection transmissions
   b) How effective are they perceived to be
   c) What are their limitations
   d) What needs to be done to improve their effectiveness?

18) What else do you think is important to improve the on-going government efforts to control Ebola Virus Disease from entering our country but we haven't yet talked about in this discussion? Probe: all responses provided

3. Key Informant Interview (KII)

1) How long have been in this position/leadership/work?

2) What do you understand about disease outbreaks? Probe for:

   - Examples of disease outbreaks
   - What outbreaks have occurred in this area?
   - What do you think was the origin of these outbreaks?

3) What can you say about Ebola disease? Probe for:

   - What are the causes of Ebola?
   - What are the main signs and symptoms?
   - How can it be transmitted?
   - How can you prevent Ebola?
   - How is Ebola treated?
   - Which are the countries that already suffered from Ebola?
   - Who do you think can get Ebola?
     i. Why do you think they can get Ebola?
     ii. Do you think there is a chance that you can get Ebola? Why and why not?

4) Normally where do people in this community seek health services when are sick? Probe for all health service sources and who are most likely to consult each?

   - How common do people in this community consult drug shops for health services?
   - What are the reasons for consulting drug shops?
   - How common do people in this community visit traditional healer?
   - What are the reasons for consulting traditional healers?
   - How common do people consult spiritual or religious healers when have health problems?
   - What are the reasons for consulting spiritual/religious healers?
   - How is the decision made on where seek health services? Who makes that decision?

5) To the best of your knowledge, just in case you encounter a person who is suspected of having Ebola like symptoms, probe for:

   - What would you do?
   - What do you think your family would do? How about other families, what will they do?

- How about the communities, what will they do?

6) To the best of your knowledge, what are the ways you know can be used to care for someone who is suspected to die due to Ebola Virus Disease in family or community? (Probe for specific actions at family level and community level)

7) What are beliefs/practices that can put someone at risk of getting Ebola? Probe for :

- Which among the beliefs/practices you mentioned above are present in your community? How can we mitigate them?
- What do you think can be done to avoid the risky beliefs?

(If that person cannot identify them, mention them, e.g. Sleeping near a highly infectious corpse for several nights, believing that doing so allows the transfer of power, believing that traditional healers can cure diseases etc.).

8) What norms, traditions and customs that you know that can put someone at risk of getting Ebola? Probe for:

- Which among the norms, customs and traditions you mention above are present in your community?
- How can we mitigate them? **(If that person cannot identify them, mention them, e.g. Burial traditions such as some mourners bathe in or anoint others with rinse water from the washing of corpses, sleeping with a corpse inside the house before burial)**.

9) What can be done to prevent Ebola from entering in your community and our country?

   Probe:
   At family level, community level and national level)
   What can you do?
   What support will you require?

10) What roles can other community members do to prevent Ebola?

- What can women do?
- What men can do?
- What can the youth do?
- What can religious leaders do?
- What can community leaders do?

11) Now think of your working conditions/environment and your colleagues, probe:

- What are some of the strategies that you know can help in the fight against disease outbreaks and Ebola to protect people with the same profession like you?
- What can you or people around you do to protect themselves, their colleagues and customers at work from getting Ebola just in case it gets into our country?

12) What are the communication channels used to communicate information on disease outbreaks and prevention in the community? Probe:
    - All (list) existing communication channels (e.g electronic – radio, TV, mobile phones; print – magazines, newspapers, posters/banners, fliers etc.; outdoor – billboards etc.; interactive – roadshows; interpersonal (door to door visits, community meetings, focus groups etc.), social media, internet-email, websites)
    - Which ones do people trust most? Why? (consider the different population groups e.g local leaders, community members, elderly, men, women, youth etc)
    - Which ones are likely to reach the majority in the community?
13) What are the key messages provided when there is or potential of disease outbreak in the community? Probe:
    - Disease signs and symptoms, risk sources, how to prevent etc
    - Who provides that information? How?
    - What do you think misses when such information is provided?
    - What should be done for such information to be useful and beneficial to the community members? Consider the different population groups

14) What are the preferred communication channels and messages to inform communities on Ebola? Probe for:
    - Messages that work well in the community, that are also cultural and language sensitive and acceptable
    - What is the best way to deliver those messages to reach the majority especially women?
    - What is the best language that can be used to reach the majority in your community? (Probe for Kiswahili or local language).
    - What communication channels are likely to reach the majority in the community? Why?
15) Who would be the best person to disseminate information about Ebola in the communities? Why? Probe:
    - Teachers
    - Health care workers
    - Community leaders,
    - Youth,
    - Women etc.)
16) What can stop people from getting clear and correct information about Ebola in your community?
    - How does that affect communication?
    - What can be done to address that?
17) Ebola virus disease can be transmitted through eating infected bush meat; contact with saliva, nasal secretions, excreta, faeces, and fomites of infected person; contact with Bats; contact with infected bush meat, mourners who have direct contact with bodies of a deceased Ebola patient, during burial rituals, attending/participating in funeral of a deceased Ebola patient and people who travel to Epidemic Areas. Now think of those means of transmission; Probe:

- Which among those are present in your community?
- How best can we educate the communities from avoiding/mitigating those cultural/practices issues?
- How can that communication to reach the majority in the communities?

18) What are the main economic activities in this community? Probe:
    - How are these activities likely to promote transmission of EVD in the community
    - How are these activities likely to challenge EVD prevention efforts? And what needs to be done to avoid that?

19) How do people in this community cope in case of disease outbreak? Probe:
- Existing community structures on prevention of infection transmissions
- How effective are they perceived to be
- What are their limitations
- What needs to be done to improve their effectiveness?

20) What else do you think is important to improve the on-going government efforts to control Ebola Virus Disease from entering our country but we haven't yet talked about in this discussion? Probe: all responses provided
